# Protein Dynamics in Solution by Quantitative Crosslinking/Mass Spectrometry

**DOI:** 10.1016/j.tibs.2018.09.003

**Published:** 2018-11

**Authors:** Zhuo A. Chen, Juri Rappsilber

**Affiliations:** 1 Bioanalytics, Institute of Biotechnology, Technische Universität Berlin, 13355 Berlin, Germany; 2 Wellcome Centre for Cell Biology, University of Edinburgh, Edinburgh EH9 3BF, UK

**Keywords:** protein dynamics, protein conformational changes, interactions, crosslinking/mass spectrometry, quantitation

## Abstract

The dynamics of protein structures and their interactions are responsible for many cellular processes. The rearrangements and interactions of proteins, which are often transient, occur in solution and may require a biological environment that is difficult to maintain in traditional structural biological approaches. Quantitative crosslinking/mass spectrometry (QCLMS) has emerged as an excellent method to fill this gap. Numerous recent applications of the technique have demonstrated that protein dynamics can now be studied in solution at sufficient resolution to gain valuable biological insights, suggesting that extending these investigations to native environments is possible. These breakthroughs have been based on the maturation of CLMS at large, and its recent fusion with quantitative proteomics. We provide here an overview of the current state of the technique, the available workflows and their applications, and remaining challenges.

## Highlights

In-solution dynamics of protein structures and their interactions can be studied by QCLMS.

Successful applications of QCLMS provide insights into multiple different biological processes.

Recent advances in QCLMS allow analyses in the context of native cellular environments, including living cells.

Alternative workflows allow researchers to tailor the analysis to their biological question.

Progress in data processing now offers this technique to researchers with limited initial expertise in crosslinking and quantitative proteomics.

## QCLMS Reveals Protein Dynamics

Molecular switches govern the assembly of protein complexes and regulate the activity of networks through irreversible domain rearrangements. Other cellular processes are controlled by alterations in interconversion rates and the relative populations of conformational ensembles of proteins. Irrespective of their exact nature, protein dynamics are influenced by their cellular environment and thus are largely intractable to traditional structural biology methods. Several novel tools are available to assist in this regard. In-cell NMR 1, 2 can follow the dynamics of proteins in cells provided that the proteins can be labelled and are not too large (<20 kDa for folded protein). Fluorescence resonance energy transfer (FRET) 3, 4, 5 and electron paramagnetic resonance (EPR) [6] circumvent protein size limitations by targeted insertion of paired donor and acceptor, or two spin labels, respectively. However, this requires a prior hypothesis and elaborate manipulation of the protein. MS-based methods using hydrogen–deuterium exchange (HDX–MS) [7] or limited proteolysis (Lip–MS) [8] allow protein conformational dynamics to be studied in complex matrices, albeit still only outside of cells. Until very recently the study of cellular processes structurally *in situ* remained highly challenging. Recent progress in QCLMS (also known as QXL–MS) is now changing this.

As part of a crosslinking analysis, proteins react with added crosslinkers under physiological conditions, which include environments such as inside living cells. The crosslinker has two reactive ends, either of which can react with an amino acid residue at the surface of a protein. A pair of residues must be sufficiently close in space to be linked. Crosslinked residue pairs (crosslinks) are then detected by MS in the form of crosslinked peptide pairs following proteolytic cleavage of the protein. Identifying the links thus provides useful information on the 3D fold of a protein; however, the resulting model is static 9, 10, 11, 12. When a protein/protein complex changes its state (e.g., conformation or composition) this also alters which residue pairs can be linked. Even if the structural changes are very small, changes in linkages or the yield of individual links may be observed. Changes in crosslinks, and thereby in structure, are detected by quantitative comparison of MS signal intensities of crosslinks that are obtained from different protein states.

**Figure 1:**
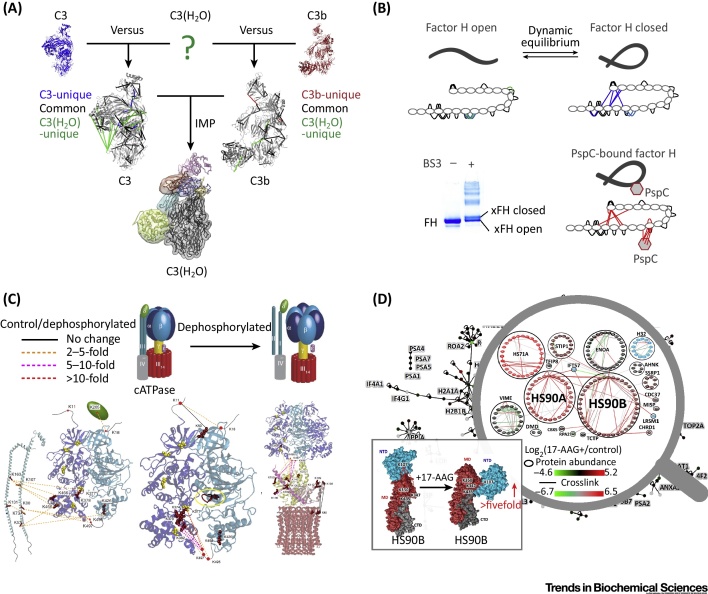
Applications of Quantitative Crosslinking–Mass Spectrometry (QCLMS). (A) Comparing complement C3(H_2_O) to inactive C3 and another active product C3b, quantitative crosslinking/MS analyses revealed structural similarities and differences between C3(H_2_O) and the other two forms of the protein. Integrative modelling utilising quantified crosslinking data with crystal structures of C3 and C3b gave rise to a structural model of C3(H_2_O). (B) QCLMS analysis of two monomeric crosslinking products of factor H (FH) revealed an open and a closed conformation of FH. Binding of pathogen protein PspC to FH shifts the equilibrium predominantly towards the closed conformation, which is inferred to promote FH-mediated suppression on complement activation. (C) Comparison of untreated and dephosphorylated ATPase revealed major changes in the yield of crosslinks between subunits, suggesting that phosphorylation regulates subunit interaction and, in turn, the function of this ATPase. (D) Combing *in vivo* crosslinking and SILAC labelling, QCLMS analysis provided a quantified protein interaction network in Hela cells, revealing changes in both protein conformations and their interaction in response to 17-AAG treatment. The magnified view shows a subnetwork involving Hsp90. A model of conformational changes of Hsp90 upon 17-AAG treatment is shown in the detailed view. The conformational change is only detected in a cellular context. Abbreviations: 17-AAG, 17-N-allylamino-17-demethoxygeldanamycin; IMP, Integrative Modelling Platform; SILAC, stable isotope-labelled amino acids. Panels (A–D) are adapted, with permission, from [16], [18], [20], and [46], respectively.

Initially, QCLMS was limited by the typically small number of crosslinks detected in CLMS experiments, as well as by the difficulty of quantifying them, because general quantitative proteomics software was not able to take crosslinked peptides into consideration. These restrictions have been largely overcome owing to recent progress, as outlined below. Consequently, following early work proving the concepts of QCLMS 13, 14, 15, the technique has now been used to study multiple different dynamic systems including the activation and regulation of protein networks 16, 17, 18, maturation of complexes [19], regulation and action of enzymes 20, 21, 22, 23, regulation of protein interactions 24, 25, and chemoresistance of a cancer cell line [26]. In these studies, induced conformational changes, dynamic structural equilibria, and protein interaction changes have been successfully revealed with the use of quantitative crosslinking data (Figure 1). Accessibility and barriers to entry have been reduced for those with little quantitative proteomics expertise as a result of progress in data processing, including automated quantitation (described in the next section). Although a handful of technical challenges remain for larger protein systems (Box 1), the approach is ready for general deployment.

Box 1 **Current Technical Challenges of QCLMS**

Crosslinking complex protein mixtures has two fundamental problems. Crosslinked peptides are difficult to detect among the many linear peptides, exacerbated by their generally low abundance, leading to poor signal patterns [68]. Once a crosslinked peptide has been fragmented in the mass spectrometer, identifying it is then complicated by the sheer number of theoretically possible peptide combinations. Neither factor is conducive to the detection of crosslinked peptides. Enrichment of crosslinked peptides can reduce sample complexity and thereby increase the chance of detecting crosslinked peptides. Crosslinked peptides differ from linear peptides in several physicochemical properties such as charge 29, 33, 69 and size [30]. This can be exploited for chromatographic enrichment of crosslinked peptides. This can also be used to enhance their detection in the mass spectrometer, for example by preferentially selecting peptides with high charge states for further analysis 29, 33. Numerous crosslinkers have been developed to carry an affinity group 43, 70, 71, 72, 73 to enrich crosslinked peptides over linear ones.

Quantitation of crosslinked peptides suffers from their generally low signal intensity. This is a consequence of the many different crosslinks that can form from any reactive residue. Furthermore, crosslinking is rarely performed to completion to avoid proteins becoming indigestible by proteases. Low signal intensity leads to problems in analysis: elution peaks contain few datapoints, isotope envelopes are incomplete, and signal/noise ratios tend to be low. Automatic peak-integration algorithms deal with such signals only with difficulty, and signal variation in replicated analysis tends to be high. Targeted analysis can improve the sensitivity and accuracy of quantitation, albeit by limiting the scale of the analysis. Using DIA methods can improve the throughput of targeted analysis. Nevertheless, the dynamic range defined by the high signal intensity of some linear peptides and the low signal intensity of many crosslinked peptides remains a challenge that may only be addressed by improving enrichment methods. It should be noted that low signal intensity also affects isobaric labelling approaches because low precursor abundance also leads to low intensity or lack of reporter ions, resulting in inaccuracy and failure in quantitation.

Quantitative crosslinking observes changes in crosslinked residue pairs (crosslinks) that tend to be supported by few or even single MS features. This is disadvantageous because individual observations can be affected by artefacts that occur during sample processing (e.g., oxidation of methionine, or N-terminal glutamate to pyroglutamate conversion) and MS analysis (e.g., interfering signals), which reduce the reproducibility of measurements. Quantitative proteomics bases its statements of protein expression changes on independent data of multiple peptides for each protein. Likewise, QCLMS should group evidence by combining multiple crosslinks for structural interpretation.

Our current understanding of how crosslink signal changes relate to the underlying protein conformational changes is based on only a few studies. It remains to be seen in broader studies how conformational changes affect crosslink yields in quantitative detail, and how crosslink changes can consequently guide structural modelling.

Alt-text: Box 1

## Quantitation Workflows

**Figure 2:**
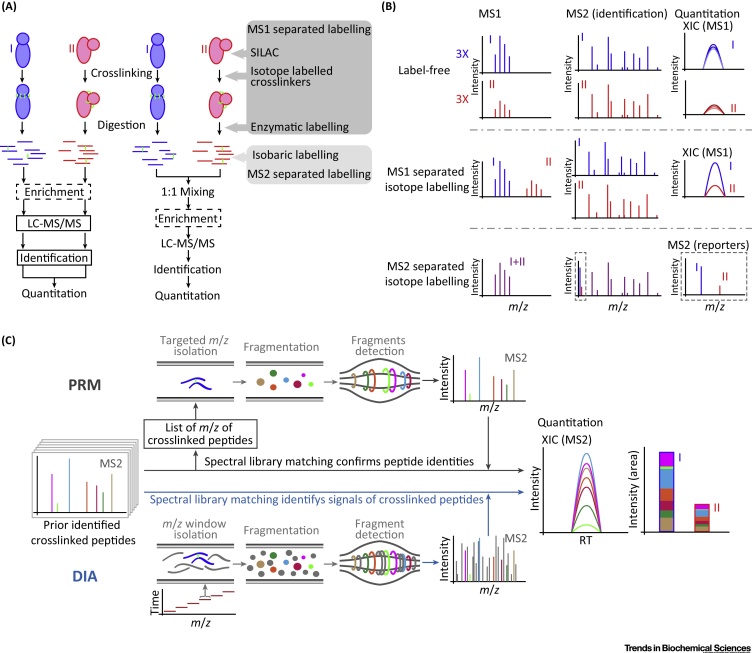
Quantitation Workflows for Quantitative Crosslinking–Mass Spectrometry (QCLMS) Analysis. (A) Overview of workflows for label-free quantitation and isotope-labelling-based quantitation. In the latter, different approaches for introducing isotope labelling are indicated in corresponding steps. (B) Strategies for LC–MS/MS acquisition and quantitation are outlined for label-free quantitation, quantitation using MS1 separated isotope labelling and MS2 separated isotope labelling. (C) Methods for LC–MS/MS analysis and quantitation are outlined for targeted quantitation using PRM and DIA. Abbreviations: DIA, data-independent acquisition; LC–MS/MS, liquid chromatography–tandem MS; *m*/*z*, mass to charge ratio; PRM, parallel reaction monitoring; RT, retention time; SILAC, stable isotope-labelled amino acids; XIC, extracted ion chromatogram.

There are six key elements that all QCLMS workflows have in common. These are crosslinking, digestion, enrichment of crosslinked peptides, liquid chromatography–tandem MS (LC–MS/MS) analysis, identification, and quantitation of crosslinked peptides (Figure 2A).

### MS1-Based Label-Free Quantitation

For label-free quantitation, different protein states are analysed as individual samples (Figure 2A). In theory, there is no upper limit to the number of conformations that can be compared. The accuracy of quantitation depends on the reproducibility of sample handling and on the use of identical amounts of each protein state, but matches that of other proteomic approaches [27].

Label-free quantitation is compatible with any crosslinking chemistry. However, it is worth noting that, in some circumstances, different protein states may only be separated after the crosslinking reaction, for example by SDS-PAGE [18]. Trypsin is usually the default choice of protease for digesting crosslinked proteins, although digestion using a set of proteases (applied each individually or sequentially) increases crosslink identifications and hence improves resolution of the CLMS analysis [28]. Because the detection of crosslinked peptides deteriorates with increasing sample complexity, numerous approaches have been developed to enrich for crosslinked peptides before LC–MS/MS analysis. When working with multiple or large proteins, crosslinked peptides can be chromatographically enriched based either on their larger charge [29] or size [30] relative to linear peptides. Both approaches have been shown to be compatible with quantitative analyses 17, 23. Affinity crosslinking reagents also have been applied for enrichment of crosslinked peptides in QCLMS analysis [26].

After digestion and optional enrichment, the peptide mixtures are analysed by LC–MS/MS typically using **data-dependent acquisition** (DDA, see Glossary), with targeted or data-independent acquisition constituting alternative approaches, as discussed below. To achieve accurate quantitation, MS1 spectra must be acquired at high resolution and with high mass accuracy. Orbitrap mass spectrometers have been the most popular choice for QCLMS analyses. Importantly, MS1 signal-based quantitation does not pose any restrictions on the type of crosslinker used (cleavable or non-cleavable), nor on the strategies for identifying crosslinked peptides (such as type of fragmentation method [31], identification based on high-resolution [32] or low-resolution [33] MS2 spectra, use of MS3 spectra [34], or multiple fragmentation methods for each precursor [35].

For the actual quantitation, MS1 signals from individual crosslinked peptides are integrated over their chromatographic elution profile, which are also called ‘extracted ion chromatograms (XICs)’ (Figure 2B). This is done separately for each crosslinked peptide and repeated for each protein state. To distinguish whether the signal change of a crosslinked peptide is likely to be due to conformational differences or to experimental variations, at least three independent crosslinking analyses (experimental replicas) are required per protein state. Most MS1-based quantitation software that can be used with crosslinking data can accommodate label-free quantitation (Box 2). Some software such as Skyline [36], MassChroQ [37], and MaxQuant 38, 39 can be used to quantify crosslinked peptides, regardless of the tools used for identification, while other software such as xTract are designed to work in conjunction with a specific identification pipeline. Owing to incomplete sampling of DDA, a crosslinked peptide is not necessarily fragmented, and therefore may not be identified in every sample despite being present. Through chromatographic alignment and matching of observed (but not necessarily identified) peaks between runs, crosslinked peptides can also be quantified in the samples where they were not initially identified. This process helps to fill in many otherwise missing values and thus improves quantitation accuracy. The specificity of matching chromatographic peaks between runs relies on high mass accuracy and good reproducibility of the chromatographic setup. The overall success of the quantitation depends crucially on robust normalisation of the intensities between runs. In addition to starting from equal sample amounts, this normalisation can be achieved by, for example, taking the signals from unaffected peptides (such as a set of common non-crosslinkable peptides) as an internal reference.

Box 2 **Software Tools for Quantitation of Crosslinking Data**

Skyline [36], MassChroQ [37], and xTract [23] are suitable or have been developed for quantifying crosslinked peptides and are freely available. They support matching MS1 features of crosslinked peptides between MS runs through chromatography alignment. XiQ [14] and MaxQuant (version 1.5.4.1 and later) [39] can quantify crosslinked peptides that are imported as a list of identified features; however, they do not support matching features between runs. mMass 41, 74 and pQuant 43, 75 have also been adapted to quantify crosslinked peptides, but only for quantitation using isotope-labelled crosslinkers. Spectronaut (Biognosis) is currently the only DIA-based quantitation software that supports the efficient input of spectra library of crosslinked peptides. Spectronaut offers an interface for visualising quantitative data, and provides a range of statistical analyses for quantitation results.

Skyline is a software tool for chromatography-based, peptide-centred quantitation. Skyline is not restricted to a particular crosslinked peptide identification pipeline. However, to quantify crosslinked peptides, the sequences of these peptides must be linearised [27]. Skyline supports label-free quantitation and isotope-labelling-based quantitation using SILAC and isotope-labelled crosslinkers [76]. Skyline has also been used for targeted quantitation of crosslinked peptides using PRM [57]. For this, MS2 data of crosslinked peptides are imported to Skyline as a transition table. From this table, Skyline generates isolation lists for constructing acquisition methods for MS, and subsequently analyses the resulting PRM data. Skyline offers a range of measurements to evaluate extracted chromatographic features, and provides an interface for visualising and correcting quantitation results. The results can be exported in .csv format for further processing and statistical analysis. Pinpoint (Thermo Fisher Scientific) works in a similar way to Skyline for MS1-based quantitation 17, 18, but requires a commercial license.

MassChroQ is another chromatography-based quantitation tool for MS data. It is different from Skyline because crosslinked peptides are imported as a list of *m*/*z* retention-time pairs. MassChroQ supports label-free quantitation and has been applied to the quantitation of crosslinked peptides using SILAC [26] and isotope-labelled crosslinkers [77]. Quantitation results can be exported in formats that are compatible with spreadsheet applications and statistical tools.

For users of the identification tools xQuest/xProphet [33], xTract is a software platform developed specifically for MS1-based quantitation of crosslinked peptides. We note that it uses a target-decoy strategy to provide statistics on the extracted ion chromatograms. xTract is capable of label-free quantitation and quantitation using isotope-labelled crosslinkers. xTract integrates programmes that summarise quantitative data from crosslinked peptide features into crosslinked residue pairs, and offers statistical analysis on signal changes of quantified crosslinks.

Alt-text: Box 2

### MS1-Based Quantitation Using Isotope Labelling

Measuring different protein states in a single MS analysis can be done by encoding the origin of the peptides through independent labelling with heavy isotopes (e.g., ^2^H, ^13^C, ^15^N, and ^18^O) (Figure 2A). The protein states are then mixed and analysed together. From the point of mixing onward, all protein states undergo identical experimental conditions, which controls for any influence from the sample preparation and analysis phases, resulting in high-accuracy measurements. So far, this approach has only been applied to binary comparisons of protein states.

Currently, isotope-labelled crosslinkers [40] are the most commonly used labelling strategy 14, 16, 17, 19, 20, 21, 22, 23, 39, 41, 42, 43, and these include a protocol for QCLMS [44]. Two different conformational states of a protein/protein complex are crosslinked separately using non-labelled and heavy isotope-labelled analogues of a crosslinker. Placing isotope labels on crosslinkers only generates differential labelling of peptides that contain a crosslinker. This minimises any increase in sample complexity. However, its application is limited to crosslinkers that are available in isotope-labelled forms.

SILAC (stable isotope-labelled amino acids) [45] is a well-established isotope-labelling approach for quantitative proteomics studies. This method can also be applied to quantitative crosslink analyses. One protein state should be prepared from SILAC-labelled material while the other should be from unlabelled material. The two protein states are crosslinked in separate tubes with the same crosslinker. This approach allows an unrestricted choice of crosslinker; however, it is only applicable to protein samples that can be labelled with SILAC. Moreover, SILAC incorporates isotope-labelled amino acids (typically lysine and arginine) into peptides, regardless of whether they are crosslinked or not. This in turn leads to a global increase in sample complexity, which further increases the difficulty of detecting crosslinked peptides. On the other hand, using such a universal labelling scheme in their study, Chavez *et al.* benefitted from this method because they were able to distinguish whether an observed crosslink change was likely due to conformational changes or simply the result of protein abundance changes 26, 46. Such normalisation for protein abundance changes is standard practice in the analysis of post-translational modifications and is essential when working with complex mixtures that are derived from different cellular states where an equal presence of the relevant protein(s) cannot be guaranteed.

In both labelling approaches, the crosslinked protein states are mixed in a 1:1 ratio, either before or after digestion. Alluding to a third strategy, Huang *et al.* [13] incorporated heavy isotopes during the trypsin digestion step. Having been crosslinked independently, one of the two protein states was digested by trypsin in normal water, while the other was digested in H_2_^18^O, which added 8 Da to all crosslinked peptides relative to digestion in normal water. The two digests were then mixed 1:1 for LC–MS/MS. One should be aware that the carbonyl oxygen exchange reaction can be difficult to optimise, which results in peptides with varying levels of incorporation and complicates the quantitation [47]. Interestingly, crosslinked peptides have two C termini while linear peptides only have one, thus both species differ in their mass shift (8 Da vs 4 Da) and can be identified by simply looking at the MS1 doublet mass difference [48]. In theory, post-digestion labelling (e.g., stable isotope dimethyl labelling [49]) is compatible with crosslinking quantitation; however, there are so far no published studies that apply the technique.

After mixing the protein states, enrichment steps, and LC–MS/MS analysis, the identification and quantitation of crosslinked peptides are conducted according to the same procedure as described in the label-free workflow. Importantly, to achieve accurate quantitation, a replicate analysis with label-swapping is required when using this procedure [17].

Within a same acquisition, isotope-labelled quantitation compares the MS signals of crosslinked peptides which are derived from different protein states (Figure 2B). This approach is therefore more accurate than label-free quantitation, as was shown in a benchmark study [23]. Isotope labelling also provides additional support for MS2-based identifications of crosslinked peptides: for example, when using isotope-labelled crosslinkers the doublet MS1 signals reveal the crosslinker, whereas when using SILAC the mass difference between the light and the heavy signals from a crosslinked peptide reveals the number of lysine and arginine residues. However, isotope labelling requires additional sample handling steps and reagents, which add to experimentation costs.

### MS2 (MS3)-Based Quantitation Using Isobaric Labelling

Alternatively, quantitation can be achieved using an isobaric labelling reagent set such as iTRAQ [50] and tandem mass tags (TMTs) [51]. Such a set of chemical reagents typically have identical masses (isobaric) and give rise to identical increases in peptide mass once attached. Each reagent consists of a reporter and a balancer. The mass of the reporter is different in every tag arising from the different distribution of stable isotopes within the molecules, and a unique reporter ion will be generated for each reagent when the labelled peptide is subjected to MS/MS. When applying isobaric labelling in a QCLMS analysis, different protein states are crosslinked and digested separately. The peptides from different protein states are then labelled individually with a different reagent. The completeness of this labelling step is of central importance to accurate quantitation. After labelling, peptides from all protein states are mixed in equal amounts and the mixture is then analysed using LC–MS/MS. In an MS1 scan, same-sequence crosslinked peptides from the different protein states are detected as a single unresolved precursor ion. Reporter ions with distinct masses are released from the isobaric reagents during fragmentation of the precursor ion. These report ion signals that are only observed in the fragmentation spectra, representing the different protein states. Relative amounts of the peptide in different protein states are quantified based on signal intensities of these reporter ions. Remaining peptide fragments (e.g., b and y ions) in a same spectrum identify the crosslinked peptide (Figure 2B). The intensities of reporter ions can be retrieved using routine peak-picking tools (such as MSconvert [52]). Isobaric labelling allows multiple protein states to be compared in a single MS acquisition, limited only by the availability of different isobaric tags. Unlike isotope labelling by crosslinkers or SILAC, peptides observed in different protein states maintain the same mass with this approach, and therefore do not increase MS1-level sample complexity. Nevertheless, a crosslinked peptide is only quantifiable if it is fragmented.

Yu *et al.* [53] reported a proof-of-principle study using TMTs for QCLMS. In their study they combined the use of MS2-cleavable crosslinkers with MS2-cleavable isobaric reagents. Following collision-induced dissociation (CID) cleavage of the crosslinker, the two liberated peptides are then individually selected for further fragmentation (MS3) and identification. The TMT reporter ions were also detected during this second cleavage step also. However, a separate synchronous precursor selection (SPS)-based MS3 scan [54] improved the accuracy of quantitation by increasing the signal intensities of reporter ions. In this way, a crosslinked peptide is identified and quantified based on up to four independent fragmentation spectra, and the results must be integrated. Thermo Scientific Proteome Discoverer from version (2.3) XlinkX node is able to handle this type of data.

### Targeted Quantitation Analysis

The three discovery-type workflows described above allow crosslinked peptides to be identified and quantitated in a single experiment. However, the accuracy of quantitation is challenged by the generally low signal intensities of crosslinked peptides (Box 1). A solution to this is targeted quantitation, which involves selectively isolating and analysing ions of known crosslinked peptides. Targeted quantitation can be based on any of the three aforementioned QCLMS workflows. Sample preparation for targeted analyses follows the same procedures as the corresponding DDA workflows. It is worth noting that all crosslinked peptides to be quantified must have been identified beforehand in a separate analysis. A targeted feature list is generated for these crosslinked peptides, instructing the mass spectrometer to filter for listed mass to charge ratio (*m*/*z*). Such a targeted approach confirmed the previous *in vitro* finding that the centres of the coiled-coils in the chromatin-resident condensin complex can also approach one another closely *in situ* in mitotic chromosomes [55]. Bruce and colleagues applied **parallel reaction monitoring** (PRM) [56] (Figure 2C) to reveal Hsp90 conformational changes upon inhibitor treatment in cells [46]. They also demonstrated that Skyline can be applied to automated targeted quantitation of crosslink data [57]. Chromatography-based quantitation requires a sufficient number of datapoints across the elution peaks of peptides, and thus only a limited number of crosslinked peptides can be monitored in each targeted analysis 56, 58.

### Data-Independent Acquisition

An alternative approach to targeted quantitation is **data-independent acquisition** (DIA; Figure 2D). Unlike targeted acquisition, it is not the MS acquisition but the data analysis that is directed towards the relevant crosslinked peptides. Reference MS2 spectra are necessary to identify the signals of crosslinked peptides in the DIA data through spectra library matching. Signals from crosslinked peptides that are derived from different protein states are compared based on their XICs from a set of user-filtered fragment ions or/and precursors. Currently, Spectronaut (Biognosis) [59] is the only software that enables DIA-based quantitation of crosslinked peptides. Recording the fragment ion signals of all detected precursors, DIA data offer maximum data coverage and reproducibility, and allow retrospective analysis. Theoretically, there is no limit to the number of crosslinked peptides that can be quantified in each analysis. However, DIA-based quantitation cannot attain the same sensitivity as PRM. In addition, false discovery rate estimation for DIA quantitation is still under discussion.

## Acquiring Residue Pair Information

Before protein structural interpretation, quantitation of experimentally measured data from crosslinked peptides needs to be consolidated into crosslinked residue pairs. Ideally, the yield of crosslinks determines the differences in MS signal between the protein states of crosslinked peptides. However, it has been shown that alternative proteolytic cleavages and post-crosslinking modifications may lead to variations in quantified signal ratios [17]. Therefore, similarly to consolidating data from peptides to proteins in standard quantitative proteomics, the signal fold-change of a crosslinked residue pair is calculated as the median value of the signal fold-change of all its supporting peptide pairs [38]. This process reduces the impact of outliers. However, compared to proteins, crosslinked residues are on average supported by much fewer datapoints. Therefore, the analysis must be rigorous in showing reproducibility between replicates. When quantifying using isotope labelling, a crosslink should be consistently quantified in replicated analysis with label swapping. In a label-free quantitation, a low coefficient of variation (CV) for replicates implies a reliable quantitation readout. A recent technical study assessing label-free quantitation of crosslinked peptides found that the reproducibility of QCLMS was in line with standard quantitative proteomics analyses [27].

## Understanding the Data

**Figure 3:**
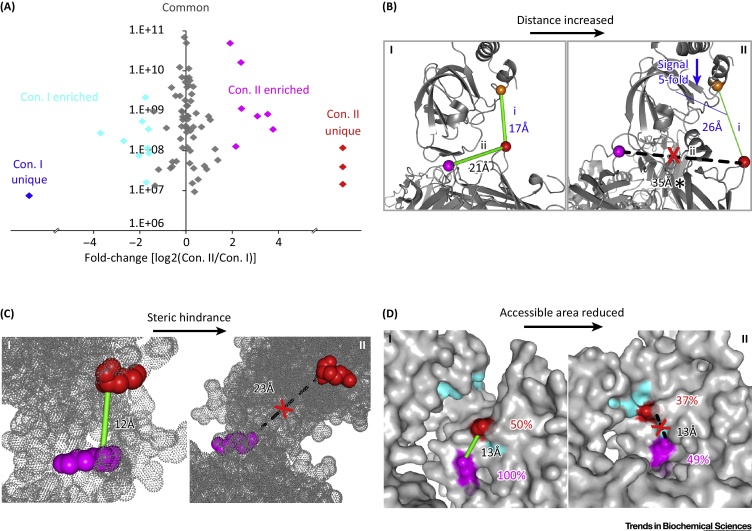
Conformational Changes Affecting the Yield of Crosslinking. (A) Typically, a binary comparison of protein states gives rise to quantified crosslinks that can fall into five subgroups. Crosslinks that are unique or enriched in either protein state reflect differences between states; crosslinks that show no changes suggest structural similarities. In the chart, the signal fold-changes of crosslinks are plotted against the observed signal intensities of crosslinks. (B) In state II, the increase in distance between crosslinked residue pair (i) co-occurs with the decrease in yield of the crosslink. The distance between residue pair (ii) becomes larger than the crosslinking limit, thus the linkage is not detected. (C) Although the pair of residues are within a crosslinkable distance in both protein states, the reduction in surface accessibility of residues prevents crosslinking in state II. (C) Distance-wise, the pair of residues can be crosslinked in both states. However, the crosslink is only observed in state I. In state II, the steric access of crosslinkers to both residues is blocked. In panels (B–D) an asterisk (*) indicates the distance between residues is beyond the limit for crosslinking. Abbreviation: Con., conformation. Panel (A) adapted, with permission, from [19]; panels (B–D) adapted, with permission, from [17].

As shown in benchmark studies, comparing the MS intensity of a crosslink obtained under different states of a protein reveals a structural change in the region of the crosslink. Quantified crosslinks can typically be divided into two groups (Figure 3A): either unique to one protein state or detected in both states. Among crosslinks that are observed in both states, some are regarded as significantly different between states. This is usually determined by an arbitrary signal fold-change cut-off or by a statistical significance test 17, 23. Most of these crosslinks exhibit fourfold or larger signal changes. When displaying quantified crosslinks in known high-resolution model protein structures, protein state-unique/enriched crosslinks tend to be concentrated in regions that are different between states. For regions that are similar between protein states, crosslinks exhibit a signal ratio close to 1:1. Major conformational changes typically lead to an absence of some cross-links and greater distance between previously crosslinked residue pairs is the most obvious cause. A crosslink is no longer detected when the distance between the pair of residues becomes larger than that which the crosslinker can bridge (Figure 3B) 16, 23, 41. In addition, crosslinks can be prevented by steric hindrance (Figure 3C) 16, 41 or if one or both residues become surface-inaccessible (Figure 3D) [16] even if the pair of residues are crosslinkable in terms of distance. Despite the changes in yield, two residues need to be accessible and remain in sufficiently close proximity in the two protein states to be crosslinked in both. In their model system, Walzthoeni *et al.* observed that enrichment for a given crosslink was consistent with changes in distance between the residue pairs as measured in the reference structural models [23]. This is not always the case, as shown in another model system [17]. Often, changes in the MS signal of a crosslink coincide with minor changes in distance, surface accessibility, and side-chain orientation in the reference structure models [17]. Therefore, although the level of change in crosslink yield seems to be related to the scope of conformational rearrangements, a quantitative correlation between the exact nature and extent of conformational changes and fold-changes of MS signals from crosslinks remains elusive.

Comparing the MS intensity of different crosslinks does not provide any structural information because their detection is heavily influenced by peptide properties during the measurement process. These properties are currently not fully understood and are not predictable. However, there are two exceptions: (i) absolute quantitation using synthetic standards [60], and (ii) comparing groups of crosslinks with each other, because averaging out will reduce individual influences from peptides [25].

## Building Structural Models

Crosslinks not only provide information on protein folding and interactions, but they also reveal regions that exhibit structural differences between conformational states. The signal fold-changes and appearance/disappearance of crosslinks in these regions allow researchers to create schematic models that elucidate the dynamic aspects of their protein systems 18, 19, 20, 21, 25, 46. Quantified crosslinking data have also been applied in integrated structure modelling and docking experiments to generate high-resolution structural models of protein states 16, 23, 46. Currently, only the constraint of distance for crosslinked residue pairs detected in individual protein states has been used. This distance constraint can be enforced during modelling or can be applied during the selection process subsequent to model generation [61]. Several software tools, such as the Integrative Modelling Platform (IMP) [62], I-TASSER [63], HADDOCK [64], ROSETTA [65], and PatchDock [66], have been used successfully (both individually and within an integrated pipeline [67]) to model crosslinking data. In comparison with identification-based crosslinking data, quantitative analysis reduces false negatives in detecting crosslinks because low-abundance crosslinked peptides may not be fragmented and identified despite being present in the samples. Quantitative analysis provides a more comprehensive list of distance constraints, which improves modelling accuracy. Signal fold-changes and lack of crosslinks in a protein state have not been considered mainly because of an ambiguity regarding the exact conformational changes.

## Concluding Remarks and Outlook

CLMS has become a standard component of integrated structural biological studies. Fusing this tool with quantitative proteomics can animate the (often) static images of protein structures generated by established technologies. QCLMS has now left the corner of method developers and benchmark studies. Although some technological challenges remain to be addressed (see Outstanding Questions), the wide set of successful applications attest to for the current breadth of research questions that can be approached. This will no doubt further expand as the technology progresses and as more application areas are explored. Importantly, QCLMS is not limited to isolated proteins or protein complexes. Screening protein structural and interaction dynamics in the context of the whole proteome is becoming possible. Therefore, both structural biology and systems biology will benefit from QCLMS, possibly assisting their fusion into structural systems biology.

Outstanding Questions How can the ambiguous information of crosslink changes (resulting either from conformation change, accessibility change, or the influence of environmental changes, or a mix of these) be disambiguated?

How can superimposed data of different conformers be deconvoluted?

How can small structural changes be detected?

What value of fold-change reliably indicates a structural change?

How can fold-change data be used in modelling?

What timescales can be captured? When does the speed of a process supersede the speed of the chemical reaction?

What is the influence of crosslinking on structural dynamics? Does crosslinking lead to shifted equilibria between multiple conformational states?

What density of crosslink data is needed to reliably call a protein structure unchanged? When does absence of signal become informative?

What influences the yield of crosslinking? Can the MS intensity of different crosslinks be used to generate structural insights?

## Glossary

**Data-dependent acquisition (DDA)**: a method for running liquid chromatography–tandem mass spectrometry (LC–MS/MS) experiments. Typically, peptides are chromatographically separated and, as they elute, peptides are directly injected into the mass spectrometer by electrospray ionisation (ESI). During MS, peptides are analysed in a repetitive acquisition cycle. In each acquisition cycle, a survey scan first detects the mass to charge ratio (*m*/*z*) and signal intensities of all ions, which are recorded in the MS1 spectrum. Among all detected ions, the top *N* abundant ions are then isolated individually and fragmented. Each isolated ion is called a precursor and its fragment ions are detected and recorded in a MS2 spectrum. The number of ions fragmented determines the duration of an acquisition cycle. For quantitative analysis, this cycle time should be controlled for such that most peptides can be analysed with over 10 acquisition cycles. High-resolution MS1 spectra enable peptide charge to be recognised, and allow charge-based precursor selection.
**Data-independent acquisition (DIA)**: an alternative method for LC–MS/MS experiments. In each acquisition cycle, the full *m*/*z* range is divided into several *m*/*z* windows. All peptides in each *m*/*z* window are isolated and fragmented together, and the resulting product ions are recorded in a MS2 spectrum. The analysis is repeated with different *m*/*z* windows until the full *m*/*z* range is covered. Optionally, a full mass range MS1 scan can be performed at the beginning of an acquisition cycle, providing information on all precursor ions. The duration of the acquisition cycle largely depends on the number of *m*/*z* windows. Peptide signals in DIA data can be identified by spectral library matching. Quantification can be carried out based on an extract ion chromatogram of fragment ions or/and precursors (when MS1 is acquired).
**Parallel reaction monitoring (PRM)**: a method for targeted quantitation using MS. In each acquisition cycle, the mass spectrometer filters for *m*/*z* values from the predefined target list, one by one. Each isolated precursor ion is then fragmented, and the resulting product ion signals are recorded in a MS2 spectrum, typically with high mass accuracy and high resolution. The duration of an acquisition cycle is determined by the number of filtered *m*/*z* values. PRM quantitation is based on the extracted ion chromatogram (XIC) of fragment ions. Even though the throughput of PRM analysis can be improved by targeting each *m*/*z* value in a narrow time-window only, the total number of *m*/*z* values that can be monitored by a PRM experiment is limited.
